# Responses of leaf functional traits to different hydrological regimes and leaf economics spectrum in the water level fluctuation zone of Three Gorges Reservoir, China

**DOI:** 10.3389/fpls.2022.939452

**Published:** 2022-09-02

**Authors:** Xiaoling Li, Di He, Gong Chen, Jin Yang, Zhengjian Yang, Xiao juan Guo, Congfeng Wang, Shijiang Zhu, Yingping Huang, Hongfeng Chen, Guiyun Huang, Dingjun Zhang, Chen Ye

**Affiliations:** ^1^Engineering Research Center of Eco-Environment in Three Gorges Reservoir Region, Ministry of Education, Hubei International Scientific and Technological Center of Ecological Conservation and Management in the Three Gorges Area, China Three Gorges University, Yichang, China; ^2^College of Biological and Pharmaceutical Science, China Three Gorges University, Yichang, China; ^3^Rare Plants Research Institute of Yangtze River, Three Gorges Corporation, Yichang, China; ^4^Key Laboratory of Aquatic Botany and Watershed Ecology, Wuhan Botanical Garden, Chinese Academy of Sciences, Wuhan, China

**Keywords:** plant functional traits, leaf economics spectrum, anti-seasonal flooding, Three Gorges Reservoir, riparian zone

## Abstract

A unique riparian ecosystem has been created as a result of anti-seasonal flooding after reservoir operations, which notably influences the distribution patterns of plant communities and their functional characteristics in the riparian zone. Plant functional traits which reflect the physiological and ecological processes of plants in particular ecosystems are crucial for indicating the variations in the ecosystem structure and function. To better understand the adaptation strategies of plants to hydrological changes and provide a scientific basis for the selection of species in the re-vegetation of the newly formed ecosystems, 14 leaf functional traits and leaf economics spectrum (LES) of 19 dominant plants under different hydrological conditions were investigated in the water level fluctuation zone (WLFZ) of the Three Gorges Reservoir (TGR). The results showed that anti-seasonal flooding has significant effects on the leaf functional traits of plants (*P* < 0.05). The net photosynthetic rate of annual plants was significantly higher than that of perennial plants (*P* < 0.05), and there was a significant correlation between leaf phenotypic and photosynthetic traits (*P* < 0.05). Canonical correspondence analysis showed that soil water content and available phosphorus were the main factors affecting the leaf function of dominant species, indicating that hydrologic factors were still important environmental factors affecting leaf functional traits of dominant species in the WLFZ. And annuals from the WLFZ have characteristics of thick leaves, high photosynthetic rate, short lifespan, and high nutrient concentrations, which make them close to the fast investment-return end of LES. On the contrary, perennials are close to the slow investment-return end of LES. The high productivity investment of annuals is better than the high defense investment of perennials for adapting to the special habitats in the WLFZ. These results indicated that different functional plants in the WLFZ of the TGR under different hydrological regimes can adopt different strategies by weighing the associations and trade-offs between their economic traits.

## Introduction

Global warming is associated with an increase in climate extremes (Jackson and Colmer, 2005) such as frequent and prolonged floods which make many ecosystems more vulnerable. Flooding is a crucial factor for triggering the dramatic alteration in composition, structure, distribution pattern, and functional traits of plant communities (Kogan, 1990; Kuroha and Ashikari, 2020; Li et al., 2020). Consequently, increasing attention has been paid to the analysis of plant functional traits and vegetation responses to environmental changes (Lamarque et al., 2014; Violle et al., 2014) in order to provide a scientific basis for predicting the spatial-temporal variation mechanism of plant leaf traits (Myers-Smith et al., 2019). Since the completion of the Three Gorges Reservoir (TGR) in 2010, the largest reservoir built, an anti-seasonal water level fluctuation zone (WLFZ) has been formed which is 175 m in winter and 145 m in summer above sea level (Shi et al., 2021). Meanwhile, the operation of “storing water in winter and discharging water in summer” and “storing clear water and discharging muddy flow” in the reservoir area has resulted in new hydrological regimes completely different from the natural Yangtze Rivers (Ren et al., 2006). The WLFZ of the TGR is characterized by frequent matter and energy exchange, low tolerance to environmental disasters, and the ecological environment is vulnerable to interference and destruction (Bao et al., 2015). In addition, the WLFZ is an important ecological safety barrier between terrestrial and aquatic ecosystems in the TGR (Chen et al., 2014). The large and frequent water level fluctuations will seriously affect the safety, health, and sustainable development of the TGR (Zeng et al., 2022). Therefore, the adaptability of plants to different hydrological regimes needs to be further studied, which would have great significance for vegetation restoration and reconstruction in the newly formed riparian forest ecosystems of the TGR and the other similar ecologically degenerative riparian areas and stream ecosystems.

A considerable body of research has shown that plant functional traits can be used to link the response of vegetation to environmental drivers such as flooding and drought (Li et al., 2018). Plant functional traits are the structural and physiological characteristics that respond to environmental variation and reflect ecosystem functions (Cornelissen et al., 2003), which can reflect the survival, growth, and reproduction strategies of plants. Plants can be closely combined with the environment, structure, and function of the ecosystem through the study of plant functional traits (Braatne and Bliss, 1999; Eviner and Chapin, 2003; Cavender-Bares and Bazzaz, 2004). As a link between plants and the environment, plant functional traits are a central topic in discussing plant ecological strategies and species coexistence mechanisms (Lugo et al., 2014). The traits that predict this relationship between environment and vegetation are known as “response traits” (Lavorel and Garnier, 2002). This is especially reflected in the response of plant traits to environmental changes, such as leaves, roots, and seeds (Diaz and Cabido, 2001; Gong et al., 2011). It should be noted that the leaf is a photosynthetic organ that has the largest contact area with the outside world and is the most sensitive to environmental stresses. Likewise, the leaf is also an important place for plants for gas exchange and water balance, which has a significant impact on the production of ecosystem substances and the global carbon cycle and water cycle (Ackerly et al., 2002; Vogelmann and Gorton, 2014). Trait-based approaches have extensively been used to predict changes in vegetation *via* plant functional traits in response to climatic conditions and hydrothermal environments to explain multiple vegetation-mediated ecosystem functions (Vendramini et al., 2002; Lavorel and Grigulis, 2012; Cheng et al., 2022). As defined by Violle et al. (2007), functional traits are morpho-physio-phenological traits that impact fitness indirectly *via* their effects on growth, reproduction, and survival, and are widely used to measure the trade-off between plant growth and resource utilization (Freschet et al., 2010). Strong evidence of plant functional traits' associations contributing to fundamental ecosystem functions has been found, especially for plant trait effects on primary production and some processes associated with carbon and nitrogen cycling (Lavorel et al., 2013). However, understanding these processes requires a profound knowledge of the complex associations and coordination between plant functional traits related to leaf/plant economics spectrums in response to environmental gradients such as flooding durations, depths, and elevations.

Plant functional traits vary in response to environmental gradients (Lavorel and Garnier, 2002; Lavorel and Grigulis, 2012). Variation of key leaf functional traits [e.g., specific leaf area (SLA), leaf total nitrogen (Nmass), leaf total phosphorus (Pmass), leaf N/P ratio (N/P), and leaf dry matter (LDM)] has been found to indicate a trade-off between a resource acquisition strategy (i.e., high values of SLA, LNC, LTN and low values of LDM) and a resource conservation strategy (i.e., high values of LDM and low values of SLA, Nmass, and Pmass). This trade-off has globally been proven to show wide-ranging and convincing evidence that feasible leaf investment strategies are to a great extent arrayed along a single spectrum, namely leaf economics spectrum (LES; Wright et al., 2004), which refers to the internal relationship between morphological structure, physiology, and biochemistry of leaves from different species. The study of plant leaf economic spectrum provides a new way to analyze the effects of global climate change on plants and their adaptation mechanism, which is a hot topic in ecological research (Chen et al., 2014; Prieto et al., 2015). Leaf functional traits at the regional scale are more closely correlated with the surrounding environment compared with the global scale (Diaz et al., 1998; Wright et al., 2001). Therefore, a comprehensive analysis of the adaptation of leaf function traits to altitude and flooding gradients in the TGR helps to better understand the adaptation mechanism and species selection to special habitat restoration in the anti-seasonal WLFZ *via* plant functional traits and the LES, which describes a nexus of interspecific correlations between these traits.

However, little is known about considering a broad spectrum of plant functional traits which might allow understanding and predicting the plant adaptation mechanism and strategies in response to environmental gradients such as altitude and flooding. In this study, we chose the mainstream and tributaries of the TGR in the Yangtze river basin from Fuling in Chongqing to Yichang in Hubei province to evaluate leaf functional traits and habitat characteristics of dominant species and construct the leaf economics spectrum in typical plant communities which would provide the theoretical basis for vegetation restoration in the WLFZ of the TGR. This study explored the following scientific issues:

(1) Plant functional traits' associations and trade-offs and their relationship with environmental gradients such as different altitudes and flooding were analyzed at the community level in the WLFZ of the TGR;
(2) Constructing the economic spectrum of plant leaves and understanding the functional characteristics of plant leaves in the WLFZ of the TGR;
(3) Exploring the plant species' responses to different hydrological rhythms and ecological adaptation strategies of plant communities in the TGR, and providing evidence for vegetation pattern and species selection in the WLFZ of the reservoir area under different hydrological gradients and the other similar riparian area and stream ecosystem.

## Materials and methods

### Natural situation of the experimental site

**Figure 1:**
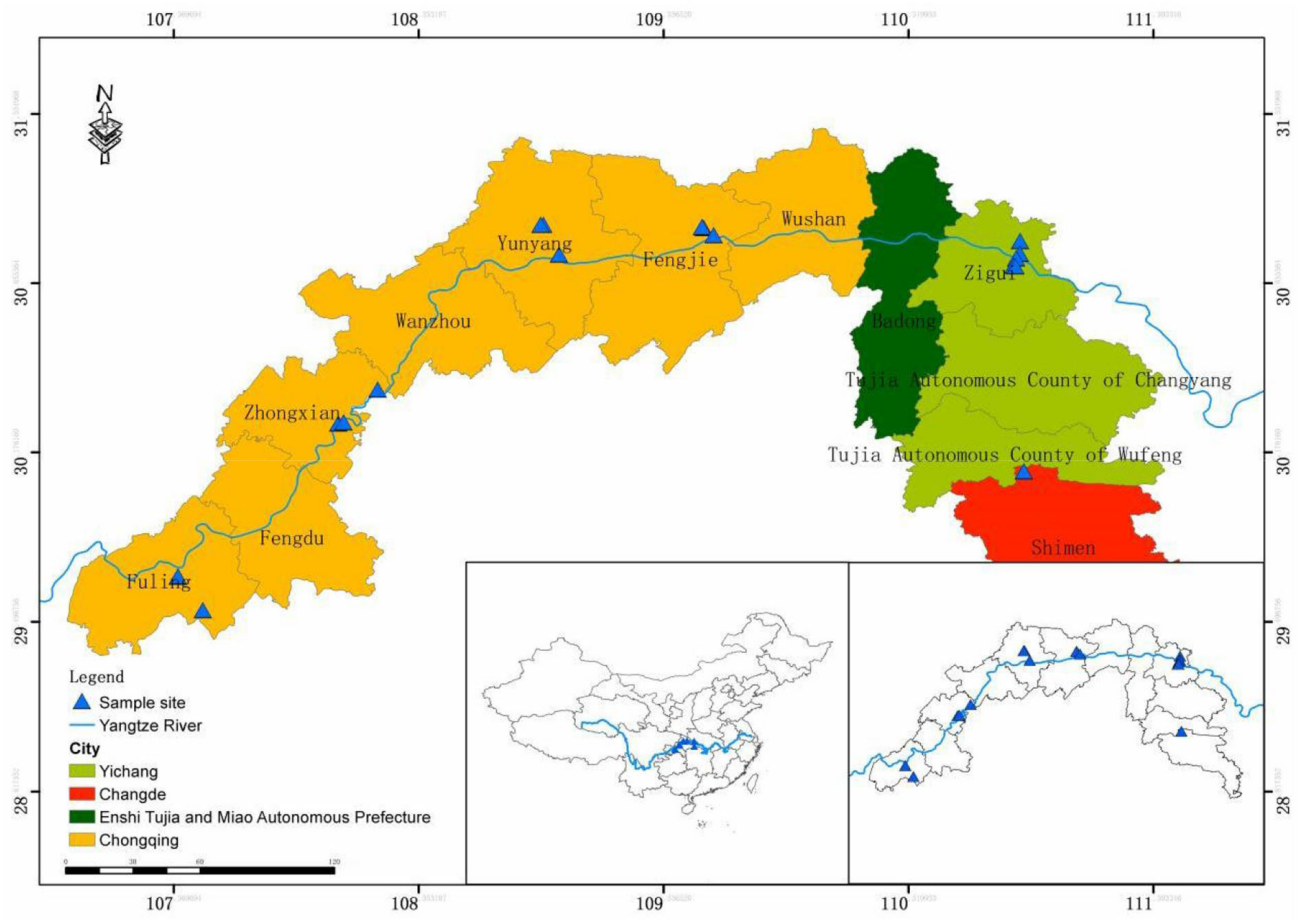
Sample point distribution map.

This study was conducted in the WLFZ (29°54′-31°09′N, 107°38′-110°75E) of the TGR (Figure 1), which extends from the Wujiang River in Fuling, Chongqing in the west to, the Xiangxi River in Yichang, Hubei is in the eastern part of China and is located in the transition zone between the subtropics and the northern subtropics. In this region, the annual mean temperature is 17.1–19.5°C, the annual active accumulated temperature is 6,500–7,000°C, the annual mean precipitation ranges from 1,100 to 1,500 mm, and the annual sunshine duration is 1,631.5 h (Chen et al., 2021).

Fieldwork was conducted from June to September 2019. Three elevations of quadrat (145–155 m; 155–165 m; 165–175 m) were set in downstream (Xiangxi River, Tongzhuang River), midstream (Tangxi River, Meixi River), and upstream (Wujiang River, the main stream of the Yangtze River in Zhongxian County) of the Three Gorges Reservoir area in China, respectively (Table 1). Among these, the Wujiang and Zhongxian plots with elevations of 155 to 175 m were investigated because plot 145 had not been exposed. Because the WLFZ was dominated by herbaceous plant communities, five quadrats with a size of 1 × 1 m were randomly set in the uniformly distributed and representative sites with each elevation. Therefore, a total of 18 sample plots and 240 quadrats were investigated. In the sample plot, 14 plant functional traits from 19 dominant plants (judged by importance value) were measured. These were leaf thickness (LT), leaf area (LA), leaf dry matter weight (LDW), specific leaf area (SLA, LA/LDW), leaf mass density (LMD, LDW/LV), leaf total nitrogen (Pmass), leaf total phosphorus (Pmass), leaf N/P ratio (N/P), water use efficiency (WUE, Pn/Tr) and photosynthetic nitrogen use efficiency (PNUE, Pn/Nmass), intercellular CO_2_ (Ci), photosynthetic rate (Pn), stomatal conductance (Gs), and transpiration rate (Tr).

**Table 1:** Sampling point details.

**Sample area**	**Sample site**	**Altitude/m**	**Longitude/°**	**Latitude/°**	**Slope/°**	**Aspect**
Wujiang river	Wujiang Bridge, Baitao Community, Baitao Town	155–175	107.4864	29.5437	27	Eastward
	Fuling District Caichang Village Caichang Tuo Goose ping village	155–175	107.3867	29.6779	21	Southeast
	Fuling district spotted bamboo forest	155–175	107.3868	29.6791	19	Southeast
Zhongxian	Shibao Village, Shibao Town	155–175	108.1886	30.4279	17	Southwest
	Zhongxian Baolin Temple	155–175	108.0305	30.2917	15	Southwest
	Dongxi Village, Dongxi Town	155–175	108.0521	30.2964	15	Southeast
Tangxi river	Yunyang County Yang Town creek village	145–175	108.9168	30.9702	28	Northwest
	Fire pulse village, Nanxi Town, Yunyang County	145–175	108.8538	31.0891	26	Northwest
	Tianya Village, Jiangkou Town, Yunyang County	145–175	108.8430	31.0896	19	Eastward
Meixi river	Fengjie County Wanian Village Meixi River bridge	145–175	109.4974	31.0768	18	Northwest
	Yuanliang Village, Fengjie County	145–175	109.4910	31.0806	16	Eastward
	Jiaochang Village, Fengjie County	145–175	109.5373	31.0481	18	Westward
Xiangxi river	Wangu Temple in Zigui County, Yichang City	145–175	110.7678	31.0240	34	Northwest
	Zigui County Inverted Tree Bay, Yichang City	145–175	110.7835	30.1001	16	Eastward
	Yichang City Zigui County eight word gate	145–175	110.7673	30.9732	24	Northeast
Tongzhuang river	Yichang City Zigui County Marine Department	145–175	110.7520	30.9545	27	southeast
	Yichang Yang Jialong Primary School zigui County	145–175	110.7418	30.9254	20	Westward
	Yichang City Zigui County Longtan River Bridge	145–175	110.7495	30.9226	18	Northeast

### Investigation of environmental factors

Upstream of the TGR, soil samples were only collected at two elevations of 155–165 and 165–175 m because the water level continued above 150 m for a long time. Using a shovel the topsoil (0–20 cm) was acquired after scraping off about 1 cm of topsoil at the sampling point, then fully mixing the soil to obtain a mixed soil sample by crushing the soil sample into large pieces, picking out gravel, animal, and plant residues, and so on. The soil samples were taken back to the laboratory for natural air drying, grinding, and screening for chemical analysis and determination. And the sampling site information was recorded and numbered. We measured the soil pH, soil organic matter (SOM), soil total nitrogen (TN), soil total phosphorus (TP), soil available phosphorus (AP), ammonium nitrogen (NH4^+^-N), nitrate nitrogen (NO3^−^-N), and soil water content (SW), using methods described by Bao (2008). Soil water content (swc) was analyzed using the drying method; altitude, longitude, and latitude were measured by GPS. Flood time (Ti) was calculated according to reservoir water level records. The slope was determined by a slope meter, and slope direction was measured using a compass. The pH value was measured by a phSJ-5 pH meter with a 5:1 soil and water ratio. Soil organic matter was determined by the K_2_Cr_2_O_7_ capacity method, while TN was measured by Semi-Micro-Kjeldahl. Ammonium nitrogen and nitrate nitrogen were determined using spectrophotometry, TP was determined by HCLO_4_-H_2_SO_4_ digestion (Molybdenum-antimony resistance colorimetric method), and AP was determined by NaHCO_3_ extraction-molybdenum-antimony resistance colorimetry. Descriptive statistics of soil environmental factors are shown in Table 2.

**Table 2:** Descriptive statistics of soil environmental factors in the water level fluctuation zone of Three Gorges Reservoir.

**Soil properties**	**Min**	**Max**	**Mean**	**SD**	**Skewness**	**Kurtosis**	**CV/%**	**K-S**
SWC	0.04	0.33	0.17	0.06	0.527	−0.704	37.37%	0.127
AP/(mg/Kg)	7.50	19.10	12.74	2.68	0.361	−0.630	21.07%	0.072
${\text{NH}}_{4^{+}}$N/(mg/Kg)	0.71	4.95	3.60	0.73	−1.372	2.794	20.17%	0.130
${\text{NO}}_{3^{-}}$-N/(mg/Kg)	6.90	19.60	12.10	2.30	0.824	0.525	19.00%	0.146
SOM/(mg/Kg)	2.28	28.63	16.59	4.60	−0.388	0.398	27.74%	0.200
PH	5.52	8.68	7.01	0.64	0.948	0.642	9.06%	0.161
TN/(g/Kg)	0.56	1.29	0.97	0.15	−0.254	−0.977	15.86%	0.103
TP/(g/Kg)	0.52	0.91	0.77	0.08	−0.569	−0.192	10.70%	0.083

Min, the minimum value; Max, the maximum value; Mean, the mean value; SD, The standard deviation; CV, Coefficient of variation; K-S, Kolmogorov-Smirnov test; SOM, soil organic matter; TN, soil total nitrogen; TP, soil total phosphorus; AP, soil available phosphorus; NH4^+^-N, ammonium nitrogen; NO3^−^-N, nitrate nitrogen; SWC, soil water content; PH, soil PH.

### Species studied

The dominating species in this study were chosen based on the important value in the sample plots at various elevations. There were 19 plant species belonging to 10 families in the WLFZ. The details of the 19 dominant species are shown in Table 3.

**Table 3:** List of tested species for leaf functional traits.

	**Species**	**Latin name**	**Family**	**Life history**	**Importance value**
1	Cocklebur	*Xanthium sibiricum*	Compositae	Annual	3.556
2	Sticktight	*Bidens pilosa*	Compositae	Annual	2.672
3	Green bristlegrass	*Setaria viridis*	Gramineae	Annual	2.772
4	Cynodon	*Cynodon dactylon*	Gramineae	Perennial	11.861
5	Yerbadetajo	*Eclipta prostrata*	Compositae	Annual	1.856
6	Humulus scandens	*Humulus scandens*	Moraceae	Annual	0.550
7	Crabgrass	*Digitaria sanguinalis*	Gramineae	Annual	2.460
8	Black nightshade	*Solanum nigrum*	Amaranthaceae	Annual	1.271
9	Water pepper	*Polygonum hydropiper*	Polygonaceae	Annual	2.369
10	Nutgrass	*Cyperus rotundus*	Cyperaceae	Perennial	3.097
11	Philoxeroides griseb	*Alternanthera philoxeroides*	Amaranthaceae	Perennial	1.659
12	Purslane	*Portulaca oleracea*	Portulacaceae	Annual	0.325
13	Eleusine indica	*Eleusine indica*	Gramineae	Annual	0.808
14	Amaranthus retroflexus	*Amaranthus retroflexus*	Amaranthaceae	Annual	0.457
15	Feather cockscomb	*Celosia argentea*	Amaranthaceae	Annual	0.080
16	Piemarker	*Abutilon theophrasti*	Malvaceae	Annual	1.726
17	Dayflower	*Commelina communis*	Commelinaceae	Annual	0.028
18	*Echinochloa crusgalli*	*Echinochloa crusgalli*	Gramineae	Annual	2.926
19	*Eriochlsa villosa*	*Eriochloa villosa*	Gramineae	Annual	0.937

### Measurements of plant functional traits

#### Photosynthetic parameters of leaves

The Li-6400 portable photosynthetic equipment (Li-cor, Inc, USA) was utilized to measure photosynthesis parameters (Pn, Gs, Tr, and Ci) in the field at 9:00–11:00 or 15:00–17:00 on a clear day to avoid midday depression of photosynthesis. Well-expanded, matured, and developed leaves at the 3rd to 6th node from shoot apices were selected for the study. An open gas channel was used and each parameter was manipulated as follows: air temperature = 25–30°C, relative humidity = 50–60%, and CO_2_ concentration = 360–380 mol m^−2^ s^−1^ (Fumiko and Fukuju, 2004). To avoid large differences in photosynthetic values of the same species, red and blue light sources were used in the determination process, and the light intensity was controlled at 1,200 μmol S^−1^ m^−2^. Photosynthetic induction was performed on the leaves first, and one value was recorded every 1 min once the photosynthetic value of leaves was stable for three consecutive periods. Three plants with similar growth patterns were chosen as test items for each species at each elevation, as well as three fully expanded and mature undamaged leaves for each plant. Following the measurement, the measured blades' markers (1–9) were collected and stored in an envelope bag (the envelope bag was labeled with sampling point information), and the labels were sorted and mailed back to the laboratory for measurement of further indicators.

#### Leaf area, dry matter weight, and specific leaf area

The unfolded leaves were collected in the laboratory in time, scanned, and saved by Canon Scan Li DE 110 scanner, and the leaf area of each leaf was calculated by Image J software (cm^2^), and the blade thickness was measured by avoiding the main vein with a spiral micrometer (Maloof et al., 2013). The scanned leaves were placed in the oven and dried to constant weight at 75°C. The dry matter weight of each leaf (g) was weighed and recorded (Precision: 0.001 g). The specific leaf area (cm^2^/g) of each plant species in various plots was calculated and recorded (Wu et al., 2019).


$$\begin{array}{l} SLA\left(cm^{2}\times g^{-1}\right)=\frac{LA\left(cm^{2}\right)}{LDW\;\left(g\right)} \end{array}$$

#### Determination of total nitrogen (Nmass) and total phosphorus (Pmass) contents in leaves

The nutritional components of the leaves were assessed in the laboratory using leaf samples obtained from the field and dried to a consistent weight. The Kjeldahl method was used to evaluate total nitrogen content in leaves, whereas the mo-Sb colorimetric method was used to determine total phosphorus level. Total nitrogen and phosphorus contents per unit weight of leaves are referred to as Nmass and Pmass, respectively (Tian et al., 2018).

### Data analysis

The significance of data from distinct water level gradient zones and different identical points was tested using a one-way analysis of variance (one-way ANOVA) in the SPSS 20.0 statistical analysis program in this experiment. The least significant range method (LSD) was employed for multiple comparisons if the difference was significant, and the significance level was set at *P* < 0.05. SPSS for Windows and Origin 8 were used for all statistical studies. Canonical correlation analysis (CCA) is a method of correlating linear relationships between two multidimensional variables (Hardoon et al., 2004). Here, the CCA was performed using Canoco 5.0 software on data from 10 environmental parameters and dominant plant leaf functional features of 10 sample plots in the Three Gorges Reservoir Area's water level fluctuation zone. Multiple environmental parameters were incorporated in the sorting process by examining the corresponding relationship between sample sequencing and object sequencing. In the same sorting graph, the outcomes of sorting samples, objects, and environmental factors can be depicted. The arrow represents environmental factors, and the arrow line represents the degree of connection between the related environmental elements and the research object in the sorting diagram. The stronger the influence on the distribution of the research object, the higher the correlation degree.

## Results

### Comparison of leaf functional traits in the upstream, midstream, and downstream of the TGR

The results showed that LT, Pn, Gs, Tr, Ci, LMD, WUE, Nmass, Pmass and PNUE had a downward trend first and then an upward trend from the upstream to the downstream contrary to LDW, LA and SLA. The leaf functional traits except for LDW and Pn of dominant species were significantly different in the upstream, midstream, and downstream of the reservoir area (*P* < 0.05; Table 4).

**Table 4:** Vertical comparison of leaf functional traits of dominant species in the fluctuating zone of Three Gorges Reservoir area (mean ± SE).

	**Upstream**	**Midstream**	**Downstream**	***df_1_***	***df_2_***	***F***	***p***
LT/(cm)	0.326 ± 0.025	0.2081 ± 0.020	0.330 ± 0.020	2	178	9.61	0.000
LDW/(g)	0.110 ± 0.021	0.137 ± 0.015	0.095 ± 0.013	2	178	1.866	0.158
LA/(cm^2^)	93.392 ± 9.234	118.867 ± 7.038	80.840 ± 6.116	2	178	5.808	0.004
Pn/(μmol.m^−2^.s^−1^)	13.326 ± 1.24	8.102 ± 0.203	10.971 ± 0.669	2	178	0.418	0.659
Gs/(mol.m^−2^.s^−1^)	0.6662 ± 0.026	0.377 ± 0.014	0.515 ± 0.056	2	178	16.093	0.000
Tr/(μmol.m^−2^.s^−1)^	17.415 ± 2.4879	11.780 ± 0.48924	13.521 ± 0.898	2	178	45.053	0.000
Ci/(μmol.m^−2^.s^−1)^	300.508 ± 12.832	123.031 ± 6.715	233.567 ± 9.471	2	178	6.15	0.003
SLA/(cm^2^/g)	846.712 ± 69.457	868.279 ± 139.745	852.747 ± 62.392	2	178	21.287	0.000
LMD/(g/cm)	0.001 ± 0.0001	0.001 ± 0.0002	0.001 ± 0.0001	2	178	71.544	0.000
WUE/(g.kg)	1.220 ± 0.125	0.822 ± 0.112	1.486 ± 0.198	2	178	6.097	0.003
Nmass/(mg/g)	3.027 ± 0.041	2.983 ± 0.022	2.992 ± 0.026	2	178	21.815	0.000
Pmass/(mg/g)	0.319 ± 0.002	0.298 ± 0.003	0.307 ± 0.002	2	178	4.132	0.018
N/P	10.072 ± 0.155	10.011 ± 0.094	9.801 ± 0.114	2	178	4.697	0.010
PNUE/(μmol.m^−2^.s^−1)^	4.403 ± 0.433	2.707 ± 0.066	3.667 ± 0.231	2	178	43.061	0.000

LT, leaf thickness; LDW, leaf dry weight; LA, leaf area; Pn, net photosynthetic rate; Gs, stomatal conductance; Tr, transpiration rate; Ci, intercellular CO_2_ concentration; SLA, specific leaf area; LMD, leaf mass density; WUE, water use efficiency; Nmass, leaf total nitrogen; Pmass, leaf total phosphorus; N/P, leaf total nitrogen to leaf total phosphorus ratio; PNUE, photosynthetic nitrogen use efficiency.

### Comparison of leaf functional traits at different elevations

**Figure 2:**
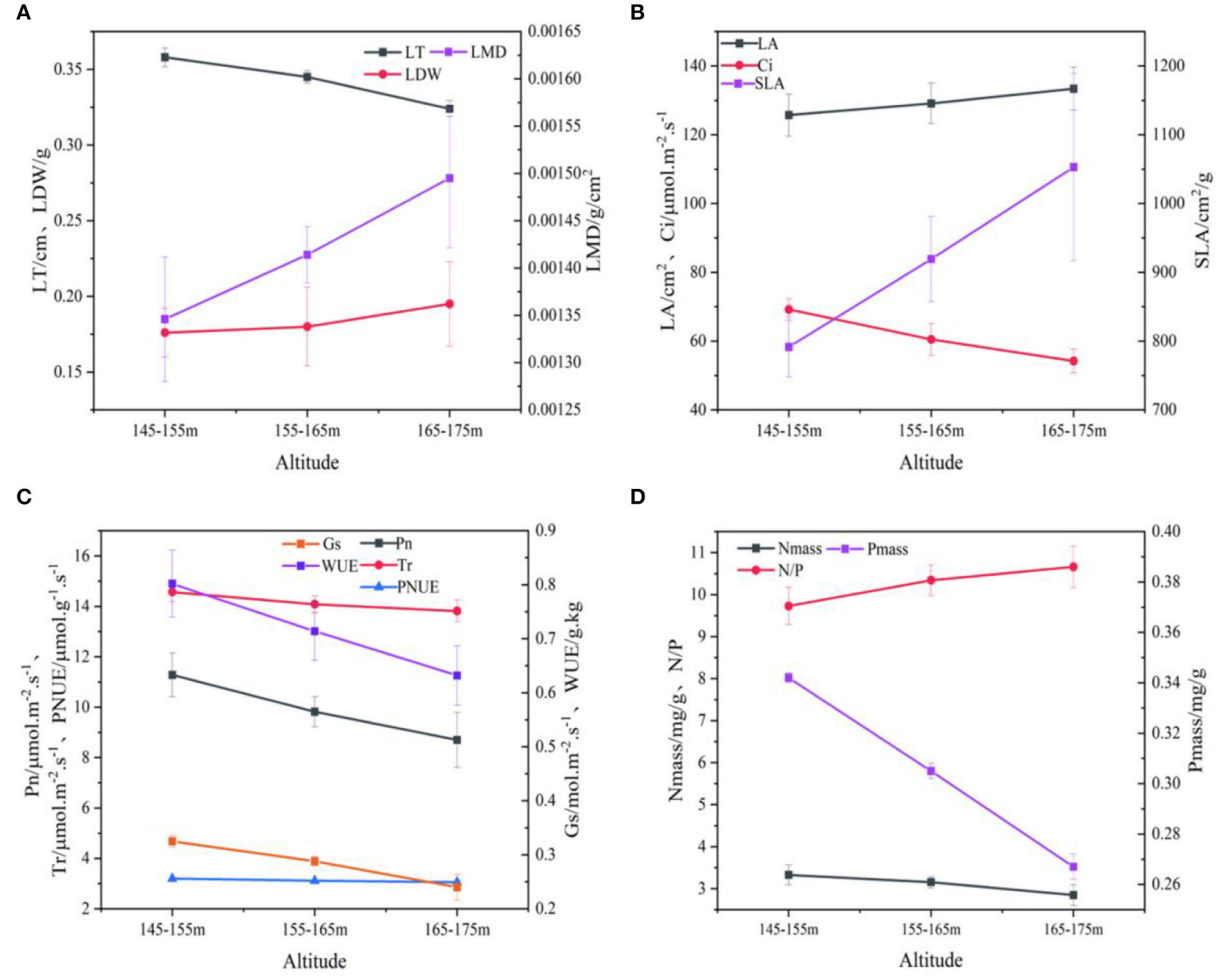
Relationship between LT, LMD, LDW and elevation gradient **(A)**. Relationship between LA, Ci, SLA and elevation gradient **(B)**. Relationship between Gs, Pn, WUE, Tr, PNUE and elevation gradient **(C)**. Relationship between Nmass, Pmass, N/P and elevation gradient **(D)**. Leaf functional properties of the fluctuating zone of Three Gorges Reservoir (mean ± SE). LT, leaf thickness; LDW, leaf dry weight; LA, leaf area; Pn, net photosynthetic rate; Gs, stomatal conductance; Tr, transpiration rate; Ci, intercellular CO_2_ concentration; SLA, specific leaf area; LMD, leaf mass density; WUE, water use efficiency; Nmass, leaf total nitrogen; Pmass, leaf total phosphorus; N/P, leaf total nitrogen to leaf total phosphorus ratio; PNUE, photosynthetic nitrogen use efficiency.

The response trends of LA, SLA, Ci, N/P, LDW and LMD in the Three Gorges Reservoir area (Figures 2A–D) were similar in the elevation gradients, and all increased significantly with elevation. LT, Pn, Gs, Tr, WUE, PNUE, Nmass and Pmass all decreased with elevation (Figures 2A,C,D). In other words, plant SLA was lower at low altitudes, but LT, leaf nutrient concentration, and photosynthetic rates were correlated with the altitude gradients, which directly reflected the adaptability of plants to the continuously changing growth environments.

### Differences in leaf functional traits among species with different life history

The LT, LDW, LA, Pn, Ci, Tr, LMD, and PNUE of the annual herbs were higher than those of the perennial herbs. There were significant differences in leaf morphological and photosynthetic features between annual and perennial plants (*P* < 0.05), although there was minimal difference in leaf nutritional content (Table 5).

**Table 5:** Leaf functional traits of plants of different life forms in the fluctuating zone of Three Gorges Reservoir (mean ± standard error).

	**Annual herb**	**Perennial herb**
LT/(cm)	0.321 ± 0.014a	0.118 ± 0.010b
LDW/(g)	0.128 ± 0.010a	0.011 ± 0.001b
LA/(cm^2^)	90.341 ± 4.553a	47.012 ± 7.894b
Nmass/(mg/g)	2.996 ± 0.019a	3.095 ± 0.046b
Pmass/(mg/g)	0.308 ± 0.002	0.309 ± 0.046
Pn/(μmol.m^−2^.s^−1^)	7.714 ± 0.620a	4.592 ± 0.525b
Gs/(mol.m^−2^.s^−1^)	1.407 ± 0.288	0.179 ± 0.031
Tr/(μmol.m^−2^.s^−1^)	10.758 ± 1.049a	3.382 ± 0.528b
Ci/(μmol.m^−2^.s^−1^)	214.975 ± 8.420	224.375 ± 17.178
SLA/(cm^2^/g)	1466.477 ± 109.881a	4445.376 ± 129.490b
LMD/(g/cm)	0.00137 ± 0.00009a	0.000303 ± 0.00003b
WUE/(g.kg)	1.129 ± 0.108a	1.787 ± 0.184b
N/P	9.779 ± 0.080	10.033 ± 0.169
PNUE/(μmol.m^−2^.s^−1^)	2.610 ± 0.214a	1.480 ± 0.166b

Different lowercase (a,b) after data in the same column indicate significant differences. LT, leaf thickness; LDW, leaf dry weight; LA, leaf area; Pn, net photosynthetic rate; Gs, stomatal conductance; Tr, transpiration rate; Ci, intercellular CO_2_ concentration; SLA, specific leaf area; LMD, leaf mass density; WUE, water use efficiency; Nmass, leaf total nitrogen; Pmass, leaf total phosphorus; N/P, leaf total nitrogen to leaf total phosphorus ratio; PNUE, photosynthetic nitrogen use efficiency.

### Relationships among leaf functional traits

**Figure 3:**
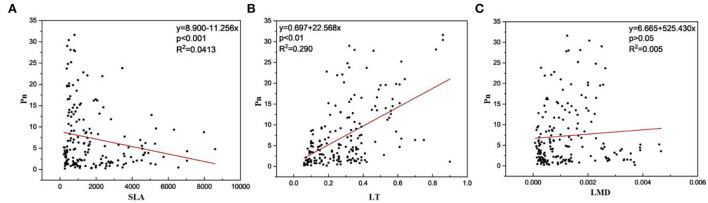
Relationship between plant leaf phenotypes and net photosynthetic rate in the fluctuating zone of the Three Gorges Reservoir area. **(A)** The relationship between specific leaf area and net photosynthetic rate. **(B)** The relationship between leaf thickness and net photosynthetic rate. **(C)** The relationship between leaf mass density and net photosynthetic rate. SLA, specific leaf area; Pn, net photosynthetic rate; LT, leaf thickness; LMD, leaf mass density.

There was also a link between photosynthetic and structural features of dominant plants in the TGR (Table 6). The LT, LDW, LA, LMD, LTN, and LTP were positively correlated with the Pn, while the SLA and N/P were negatively correlated with the Pn. And the LT, LDW, and LA had a strong correlation with the Pn, PNUE, and Tr. Meanwhile, the result of fitting leaf phenotypic traits with leaf net photosynthetic rate showed that the LT had a very significant positive correlation with the Pn (*P* < 0.01; Figure 3), and the SLA was negatively correlated with the Pn (*P* < 0.01), but there was no significant correlation between LMD and Pn (*P* > 0.05).

**Table 6:** Correlation between photosynthetic and structural characters of leaves.

	**LT/(cm)**	**LDW/(g)**	**LA/(cm^2^)**	**SLA/(cm^2^/g)**	**LMD/(g/cm)**	**Nmass/(mg/g)**	**Pmass/(mg/g)**	**N/P**
Pn/(μmol.m^−2^.s^−1^)	0.539	0.407	0.438	−0.188	0.074	0.105	0.195	−0.048
Gs/(mol.m^−2^.s^−1^)	0.379	0.301	0.298	−0.179	0.104	0.119	0.083	−0.029
Tr/(μmol.m^−2^.s^−1^)	0.419	0.44	0.396	−0.286	0.177	0.051	0.118	0.041
Ci/(μmol.m^−2^.s^−1^)	0.243	0.002	0.146	0.075	−0.225	0.128	0.29	0.095
WUE/(g/kg)	−0.07	−0.251	−0.141	0.212	−0.278	−0.008	0.052	−0.042
PNUE/(μmol.m^−2^.s^−1^)	0.551	0.424	0.452	−0.202	0.082	0.192	0.195	−0.022

P < 0.5, significant correlation; p > 0.1, extremely significant correlation.

LT, leaf thickness; LDW, leaf dry weight; LA, leaf area; Pn, net photosynthetic rate; Gs, stomatal conductance; Tr, transpiration rate; Ci, intercellular CO_2_ concentration; SLA, specific leaf area; LMD, leaf mass density; WUE, water use efficiency; Nmass, leaf total nitrogen; Pmass, leaf total phosphorus; N/P, leaf total nitrogen to leaf total phosphorus ratio; PNUE, photosynthetic nitrogen use efficiency.

### Response of leaf functional traits to environmental factors

**Figure 4:**
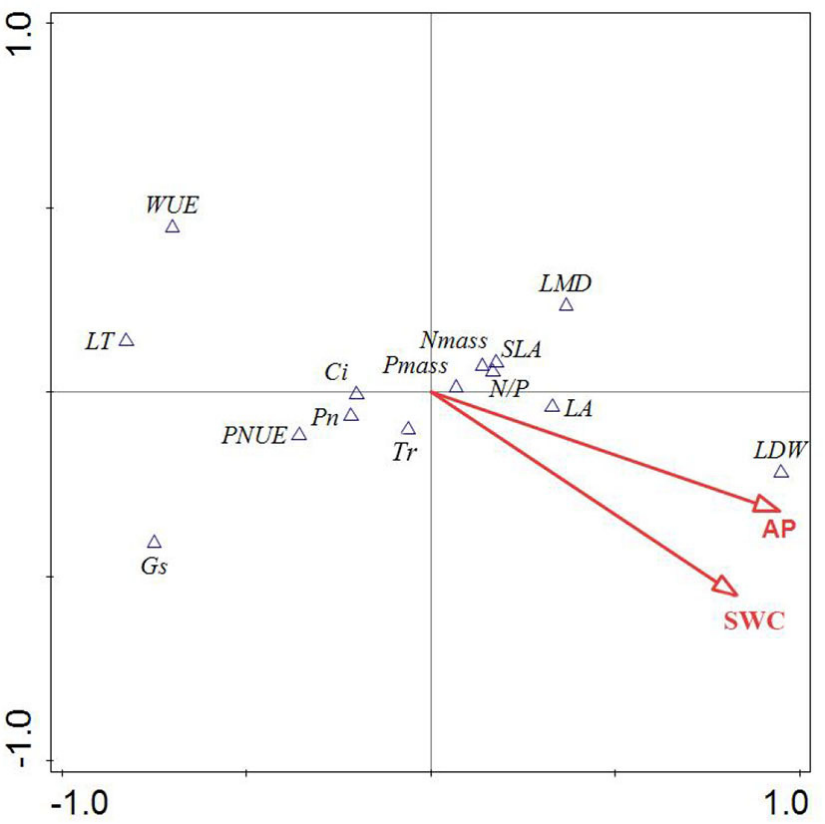
CCA sequence of leaf functional properties and environmental factors in the water level fluctuation zone of Three Gorges Reservoir. Because SWC and AP have the highest explanatory degree for leaf functional traits of plants, only SWC and AP are selected for the diagram to highlight the differences explicitly. LT, leaf thickness; LDW, leaf dry weight; LA, leaf area; Pn, net photosynthetic rate; Gs, stomatal conductance; Tr, transpiration rate; Ci, intercellular CO_2_ concentration; SLA, specific leaf area; LMD, leaf mass density; WUE, water use efficiency; Nmass, leaf total nitrogen; Pmass, leaf total phosphorus; N/P, leaf total nitrogen to leaf total phosphorus ratio; PNUE, photosynthetic nitrogen use efficiency; AP, soil available phosphorus; SWC, soil water content.

The CCA analysis was conducted on leaf functional traits and environmental factors of dominant species in the TGR (Figure 4). The CCA results showed that the first and second ranking axes explained 86.56% and 13.44% of leaf functional traits variables, respectively. Therefore, data from the first two axes were used to analyze the relationship between leaf functional traits and environmental factors. Monte Carlo random displacement test also showed that all canonical characteristic axes were significantly correlated with leaf functional traits (*P* < 0.05), indicating that CCA ranking results were reliable. Only soil water content (SWC) and soil available phosphorus (AP) could explain the leaf functional features of dominating species in the reservoir region, according to the ordination diagram, and AP and SWC were the primary influencing factors on LDW, LA, SLA, LMD, Pmass, Nmass, and N/P. The impact of AP and SWC on LDW was more substantial when leaf characteristics were projected.

## Discussion

### Analysis of the position of plants with different life histories on the leaf economics spectrum

The integration of direct and indirect associations and trade-offs among leaf functional traits can be reflected in the leaf economics spectrum. Species of different life forms occupy different positions in the leaf economic spectrum for adapting to habitats (Pan et al., 2020). The study of the plant leaf economics spectrum offers a fresh perspective on the influence of global climate change on plants and their adaptation mechanisms, which is a popular topic in ecological research (Chen et al., 2014; Prieto et al., 2015). The commencement of operations of the TGR in 2003 triggered dramatic shifts in flooding regimes that have had important and ongoing implications for the ecological functioning of riparian ecosystems along the Yangtze river. In the present study, the characteristics of the terrestrial vegetation community have been altered substantially since submergence, with annual plants and perennials accounting for 71.23% and 27.39%, respectively. The biggest change was in the woody plant with 1.37% of the total number of species. Annual plants showed stronger growth with high morphological and photosynthetic characteristics than perennials contributing to adaptation to different hydrological gradients, which is consistent with previous research findings (Tilman, 1988; Mao et al., 2012, 2014). The reason may be that plants from different life forms have different environmental adaptation strategies. The community structure of the WLFZ of the TGR would tend to be simple because of the long-term anti-seasonal flooding and the increasing frequency of sudden and sporadic aridity in the Three Gorges Reservoir region.

Herbaceous plants have advantages in the WLFZ because they frequently are at the quick-return end of the economics spectrum with high nutrient concentration, high photosynthetic rate, fast respiration rate, and short lifespan compared with shrubs and trees (Jie et al., 2012; Yu et al., 2014; Huang et al., 2017). In this way, herbaceous plants can complete their growth history in the short emerging time of the WLFZ so that they can avoid long-term flooding by seed dormancy after the storage of water in winter and have better adaptability to the flooding stress in the WLFZ. Annuals germinate in spring and fructify in autumn during the short exposure time of the WLFZ of the TGR. So, the phenology of these adaptive annual plants does not compound with the submergence occurring time, and able to survive as dominant species. Perennial plants need a longer period to complete their full life cycle and the short exposure time of the WLFZ is not sufficient for perennials vegetation to complete the seed-to-seed life cycle. Herbaceous plants, especially annual herbaceous plants, can better adapt to the unique habitats in the WLFZ of the TGR because annual herbaceous plants have strong environmental adaptability and fitting life span.

Annual plants were distributed in the low-altitude area, which is more disturbed by summer flooding and long-term anti-seasonal flooding in winter, and the special environmental pressures triggered associations and trade-offs between leaf functional traits of annual plants for survival and growth under the alternative wet and dry environments with severe disturbances and poor nutrients. Annual plants were characterized by low specific leaf area and thick cell walls which can reduce water loss and protect plants from high-intensity light radiation brought by exposed water fluctuation zone, to adapt to the harsh environment of low altitudes with higher interference intensity from flooding in the reservoir area (Tsialtas, 2004). This further proved that the characteristics of thick leaves, small leaf area, small specific leaf area, and strong photosynthetic capacity of annual herbs are more inclined to adopt the “quick return” growth strategy. Leaf economics spectrum can be used to explain a series of ecological adaptive functional traits of plants with different life histories in response to environmental gradients. Annuals were near the quick invest-return end of the leaf economics spectrum that is characterized by thick leaves, high photosynthetic rates, and a short lifespan. On the contrary, perennials were at the slow invest-return end of the leaf economics spectrum.

### Combination characteristics of plant leaf traits based on leaf economics spectrum

There was also a certain correlation between the functional traits in plants' life history strategy and survival abilities (Kawai and Okada, 2020). The optimal functional combination among traits is formed by the synergistic changes among traits in plants, to achieve a good adaptation to the constantly changing external environment (Jeffrey et al., 2014; Mello et al., 2020). In the correlation analysis of plant leaf structure and photosynthetic traits, the results showed that LT, LA, LDW, LMD, LTN, and LTP were positively correlated with Pn, while SLA and N/P were negatively correlated with Pn. The relationships also confirmed that the plants were able to “invest” resources by putting photosynthates and mineral nutrients into the construction of leaf structure, and plant leaves in turn provided an income stream of photosynthates. This kind of investment in plants through photosynthesis is sustained throughout the whole life period, namely, the photosynthesis of plants can maintain leaf growth and reinvest in it, and provide a source of energy for stems and other parts of plants. This investment pays off throughout the life of the plant through photosynthesis, which allows the plant to sustain and reinvest in its leaves and provide energy for the stem and other parts of the plant (Weraduwage et al., 2016; Li et al., 2019). Specific leaf areas which reflected the light capture and nutrient utilization efficiency of leaves can affect photosynthesis and other abilities of plants (Kunstler et al., 2015). Because they require more dry matter in defense construction to protect against adverse environmental circumstances, plants that live in relatively poor environments or at the early stages of succession have smaller SLA. Plants use dry matter in their leaves to build guard cell structure, mesophyll cell density, and leaf thickness, which can accommodate more chloroplasts, reduce water loss, and boost photosynthetic capacity (Wilson et al., 1999; Garnier et al., 2001). A proof of plant convergent evolution is the association between several features based on the leaf economics spectrum (Grime, 2006; Lai et al., 2021). Nitrogen and phosphorus are indispensable nutrients for the photosynthesis of plants, and plants are sensitive to nitrogen supply in the photosynthesis of plant leaves (Milroy and Bange, 2003). This is because chlorophyll content, Rubisco, and enzyme activity are related to photosynthesis (He et al., 2006). Phosphorus is an integral component of many important organic substances, such as energy, and it has a direct effect on photosynthesis (Zurano et al., 2021). At different scales, the associations among leaf functional traits were not equal, and it was unclear how much variation in one trait influenced the response of other traits to altitude (Royer et al., 2014).

### Response of leaf functional traits to the environment based on leaf economics spectrum

Plant functional traits can reflect the adaptation of plants to the changes in biotic and abiotic environments (Cheng et al., 2022). In this study, the leaf functional traits of dominant species in the WLFZ were different in the upstream, midstream and downstream of the TGR, and there were significant differences in leaf functional traits in addition to leaf dry weight and net photosynthesis. This result indicated that the distance from the reservoir may also play a role in influencing how plant functional features respond to hydrological regimes. Different distances may result in changes in topography, flow velocity, and soil properties, all of which affect vegetation development and consequently have a substantial impact on vegetation functional traits (Ye et al., 2020). There were differences in photosynthetic rate and leaf dry weight in different geographical situations mainly due to temperature differences and light intensity (Wright et al., 2017). However, it was found that the temperature difference between the upper, middle, and lower reaches of the Three Gorges Reservoir was minimal; they were 22, 24, and 23°C, respectively. The light intensity was also similar upstream, midstream, and downstream. Therefore, the net photosynthetic rate and leaf dry weight may not be significantly affected.

Leaf functional traits were affected not only by light and temperature but also by hydrological conditions (Richards et al., 2021). The present results showed that LA, SLA, LDW, and LMD of plant leaves increased with an increase in elevation, while LT decreased on the contrary. It is because different elevation gradients in the WLFZ meant different flooding depths and durations, and low elevation areas were subjected to a longer and higher frequency of flooding, while high elevation areas were less subjected to flooding stresses. As an important indicator of plant resource acquisition and survival strategy, SLA is closely related to plant growth rate and can reflect the balance between carbon acquisition and utilization of plants (Cleiton et al., 2020). Lower SLA reduces respiratory carbon loss per unit leaf area and increases carbon harvest by extending leaf life, while lower growth rates maintain a positive carbon balance, enabling low-altitude plants to survive adverse environments (Li et al., 2018). To adapt to the more barren environment and complete their life cycle in the WLFZ with a shorter emerging period, the plants at low altitudes devote more resources to produce, so the LA, SLA, LDW, and LMD are smaller than those of the plants at high altitudes. Harsh conditions increase with elevation, so high-altitude plants invest more in structure. At the same time, the photosynthetic rate of high-altitude plants was lower than that of plants in low-altitude areas, which is consistent with the results of Guan et al. (2020). This indicated that plants at low altitudes synthesize enough organic matter in a short time with a high photosynthetic rate, which can be used to compensate for leaf construction consumption. Furthermore, the effects of different water level rhythms on plants in the reservoir area were noticed (Heilmeier, 2019). Accordingly, stomatal conductance, transpiration rate, intercellular CO_2_, and water use efficiency, which are closely related to photosynthesis, decreased with the decrease of photosynthesis.

The observed increase in N/P with elevation is consistent with the strategy of quick resource acquisition and quick return in the short life span at low altitudes. According to Gusewell's (2004) threshold hypothesis, N:P<10 indicates that the growth of plants is limited by N; N:P>20 indicates that the growth of plants is limited by P;10<N:P<20 indicates that plant growth is jointly restricted by N and P. The investigation results of this study showed that plant growth in the water level fluctuation zone was jointly restricted by N and P. The results showed that the contents of N (3.09 mg·g^−1^) and P (0.31 mg·g^−1^) in leaves of plants were much lower than the geometric mean values of N and P in leaves of land plants (*N* = 18.74 mg·g^−1^, *P* = 1.21 mg·g^−1^). The N and P contents of plant leaves are usually closely related to the N and P contents of the soil. Unnatural flooding destroys the biogeochemical process and leads to the gradual depletion of soil nutrients, which indirectly leads to the reduction of vegetation nutrients in the WLFZ.

The link between leaf functional traits and environmental factors can be readily seen in the CCA analysis, which showed that among various environmental factors in the reservoir area, AP and SWC had the greatest influence on leaf functional traits of dominant species, and AP and SWC were the main influencing factors on leaf functional traits such as LDW, LA, SLA, Nmass, Pmass, and N/P, with a more significant effect on LDW. It indicated that soil physical and chemical properties in the water level fluctuation zone were affected by the fluctuation of water level, and soil moisture and nutrients are important factors for plant growth. With the increase in altitude, the flooding time in the water level fluctuation zone decreased correspondingly, and the accompanying changes in soil water made plants form new adaptive strategies by adjusting leaf functional traits (Kunstler et al., 2015). Chinese vegetation is more susceptible to the limitation of P content in the soil, which is considered a major constraint on plant productivity because it regulates molecular to ecosystem biological processes in many terrestrial ecosystems, according to studies of nitrogen and phosphorus content in leaves (Shanjia et al., 2021). Therefore, SWC and AP play a leading role in the formation of plant functional traits in the WFLZ of the TGR, although the functional traits are the result of a combination of multiple environmental factors.

### Leaf economics spectrum of plants in the TGR

**Figure 5:**
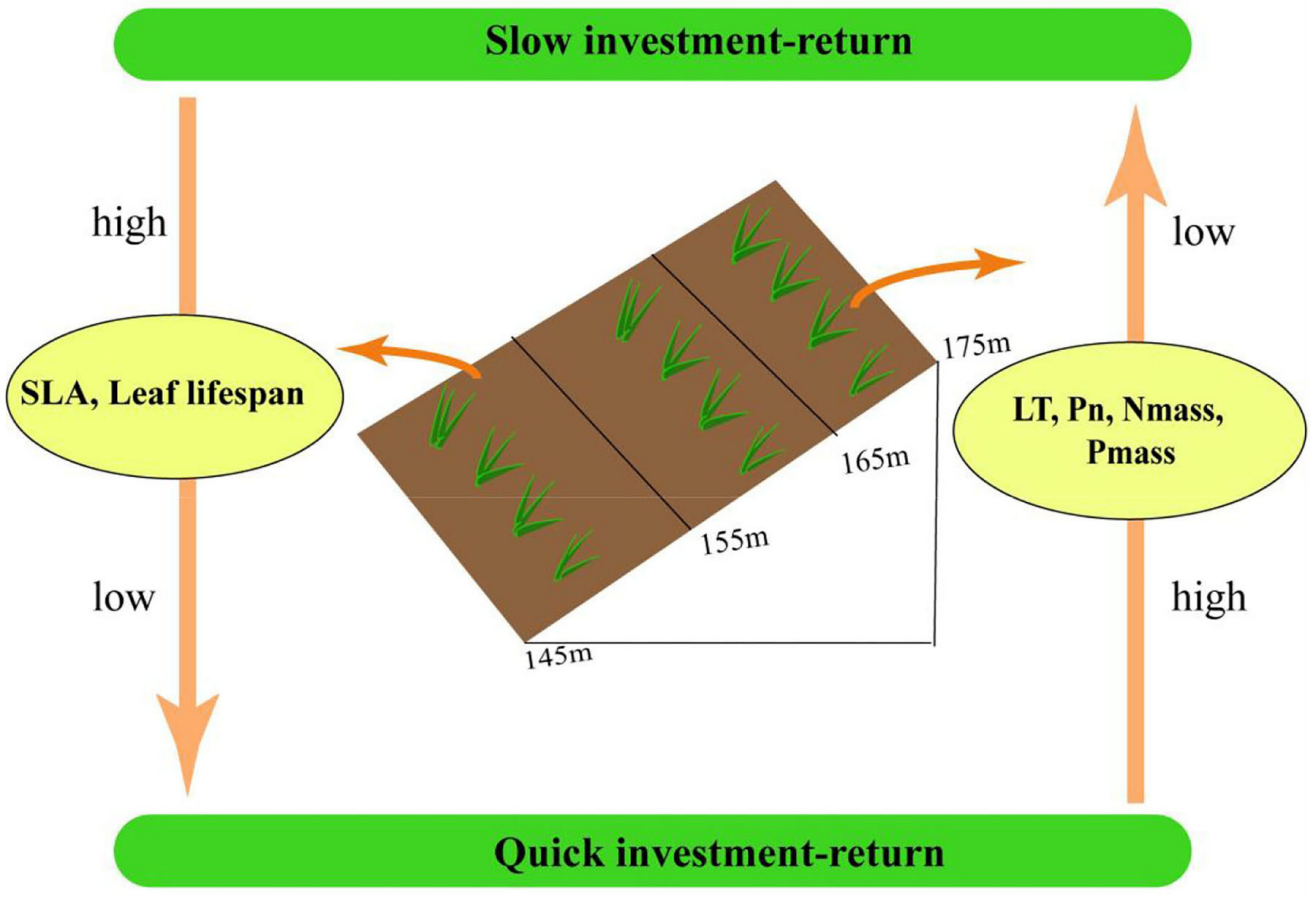
Concept map of leaf economics pattern spectrum in water level fluctuation zone of Three Gorges Reservoir area. SLA, specific leaf area; LT, leaf thickness; Pn, net photosynthetic rate; Nmass, leaf total nitrogen; Pmass, leaf total phosphorus.

This study not only distinctly demonstrated the associations and trade-offs between leaf functional traits, but also further confirmed the leaf economics spectrum advanced by Wright et al. (2004) to quantify the trade-offs among leaf traits and their variation patterns on a global scale. The leaf economics spectrum represents the resource acquisition-utilization trade-off strategy of plants. In the WFLZ of the TGR, annual plants are typical quick investment-return species, while perennial plants are typical slow investment-return species (Figure 5). The position of plants in the leaf economics spectrum also indicates that plants can adjust their structure and physiological traits to adapt to different environmental gradients, and the emergence of leaf economics spectrum also provides a new perspective to explore the adaptability of plants.

## Conclusion

(1) Herbaceous plants dominated the plant community in the WLFZ of the TGR, and annuals were significantly more than perennials. In the low altitude with a barren environment, annuals adopted the “quick return” investment strategy, and the photosynthetic capacity of plants increased significantly.
(2) The spatial heterogeneity of the water level fluctuation zone increased with the increase of the altitude gradients, and the plants also made some adaptive changes to the leaf functional traits. The results showed that the annual herbaceous plants were more distributed in the low altitude area and were more adapted to the characteristics of the low altitude area than the perennial herbaceous plants.
(3) Annuals from the WLFZ have characteristics of thick leaves, high photosynthetic rate, short lifespan, and high nutrient concentration, which make them close to the fast investment-return of the leaf economics spectrum. On the contrary, perennials are close to the slow investment-return end of the leaf economics spectrum. The high productivity investment of annuals is better than the high defense investment of perennials for adapting to the special habitat in the WLFZ.

This study explored the changes in leaf economic traits of dominant species in different habitats in the Three Gorges Reservoir area and revealed the response strategies of leaf functional traits to the hydrological gradients for survival and growth of plants in the anti-seasonal fluctuation zone. It has great significance to the vegetation restoration and reconstruction in the WFLZ of the TGR. It is suggested that the general policy of restoring and constructing artificial communities in the reservoir area should be mainly herbaceous, supplemented by shrubs or small trees. However, this paper did not study the changes of the same species at different altitudes and samples, as well as the differences and relationships among different plants (such as different families and photosynthetic plants). In addition, the soil environment and vegetation in the WFLZ of the TGR are always in dynamic change, so it is necessary to continue to monitor and study the spatial-temporal changes of plant characteristics and environmental factors to better understand the variations in the characteristics of the leaf economics spectrum of vegetation in the reservoir area.

## Data availability statement

The raw data supporting the conclusions of this article will be made available by the authors, without undue reservation.

## Author contributions

XL, DH, and ZY contributed the central idea, analyzed most of the data, and wrote the initial draft of the paper. GC, JY, XG, CW, SZ, YH, HC, GH, DZ, and CY contributed to refining the ideas, carrying out additional analyses, and finalizing this paper. All authors contributed to the article and approved the submitted version.

## Funding

This work was supported by the National Natural Science Foundation of China (Nos. 51779127 and 51209122), partially funded by National 111 Intellectual Base (D20015), the Open Research Program of the Engineering Research Center of Eco-environment in the Three Gorges Reservoir Region of the Ministry of Education (No. KF2018-02). Open Project fund of Key Laboratory of Aquatic Plants and Watershed Ecology, Chinese Academy of Sciences (E0520204), the Youth Innovation Promotion Association of the Chinese Academy of Sciences (No. 2019334), and Research Project on Seed Preservation Technology and Facilities of Rare Plants in the Three Gorges Reservoir Area-Investigation and Collection of Flooding-Tolerant Germplasm Resources (SDHZ2021346).

## Conflict of interest

Authors GH and DZ are employed by Three Gorges Corporation. The remaining authors declare that the research was conducted in the absence of any commercial or financial relationships that could be construed as a potential conflict of interest.

## Publisher's note

All claims expressed in this article are solely those of the authors and do not necessarily represent those of their affiliated organizations, or those of the publisher, the editors and the reviewers. Any product that may be evaluated in this article, or claim that may be made by its manufacturer, is not guaranteed or endorsed by the publisher.
